# A conjoined universal helper epitope can unveil antitumor effects of a neoantigen vaccine targeting an MHC class I-restricted neoepitope

**DOI:** 10.1038/s41541-020-00273-5

**Published:** 2021-01-18

**Authors:** Adam M. Swartz, Kendra L. Congdon, Smita K. Nair, Qi-Jing Li, James E. Herndon, Carter M. Suryadevara, Katherine A. Riccione, Gary E. Archer, Pamela K. Norberg, Luis A. Sanchez-Perez, John H. Sampson

**Affiliations:** 1 Preston Robert Tisch Brain Tumor Center, Duke University Medical Center, Durham, NC USA; 2 Department of Neurosurgery, Duke University Medical Center, Durham, NC USA; 3 Department of Pathology, Duke University Medical Center, Durham, NC USA; 4 Department of Surgery, Duke University Medical Center, Durham, NC USA; 5 Department of Immunology, Duke University Medical Center, Durham, NC USA; 6 Department of Biostatistics and Bioinformatics, Duke University Medical Center, Durham, NC USA

**Keywords:** Peptide vaccines, Cancer immunotherapy

## Abstract

Personalized cancer vaccines targeting neoantigens arising from somatic missense mutations are currently being evaluated for the treatment of various cancers due to their potential to elicit a multivalent, tumor-specific immune response. Several cancers express a low number of neoantigens; in these cases, ensuring the immunotherapeutic potential of each neoantigen-derived epitope (neoepitope) is crucial. In this study, we discovered that therapeutic vaccines targeting immunodominant major histocompatibility complex (MHC) I-restricted neoepitopes require a conjoined helper epitope in order to induce a cytotoxic, neoepitope-specific CD8+ T-cell response. Furthermore, we show that the universally immunogenic helper epitope P30 can fulfill this requisite helper function. Remarkably, conjoined P30 was able to unveil immune and antitumor responses to subdominant MHC I-restricted neoepitopes that were, otherwise, poorly immunogenic. Together, these data provide key insights into effective neoantigen vaccine design and demonstrate a translatable strategy using a universal helper epitope that can improve therapeutic responses to MHC I-restricted neoepitopes.

## Introduction

T-cell-based immunotherapy is currently being explored for the treatment of various cancers, considering the molecular-guided precision and high cytotoxic potential of T cells. There are several approaches for producing a cytolytic, tumor-focused T-cell response within a host, such as cancer vaccines and adoptive T-cell therapies (ACTs). ACTs represent a highly potent form of T-cell therapy owing to the sheer number of tumor-specific T cells that can be generated ex vivo; however, their use is currently reserved for cancers that possess at least one highly conserved (i.e., similar from patient to patient) tumor antigen or those that exhibit a high degree of T-cell infiltration. For tumor types that lack these features, cancer vaccines offer one of the few feasible options for T-cell-based immunotherapy.

Cancer vaccines work by administering, into a host, immunogens derived from tumor-associated antigens in an effort to activate endogenous T cells reactive toward these antigens. The advent of next-generation sequencing has enabled a comprehensive assessment of antigens expressed by a given tumor, offering numerous immunotherapeutic targets for vaccine strategies. Among these approaches, vaccines targeting neoantigens that arise from tumor-specific somatic missense mutations have garnered significant interest in the clinic^1–6^ owing to their ability to induce a multivalent, tumor-focused immune response. Some tumor types, such as newly diagnosed glioblastoma multiforme (GBM), possess an intrinsically low somatic mutation load^7^, providing only a limited number of potential immunotherapeutic targets for neoantigen vaccines. For these tumor types, identifying methods that can maximize the immunotherapeutic capabilities of each neoantigen will be crucial for the success of this approach.

Major histocompatibility complex (MHC) class I-restricted epitopes spanning the mutated region of neoantigens (i.e., neoepitopes) have been preferred targets in clinical studies, given the well-described role of cytotoxic CD8+ T cells in the antitumor response and the expression of MHC I on tumor cells. A common first step in the development of neoantigen vaccines is prioritizing candidate neoepitopes with a high likelihood binding to MHC class I molecules using in silico MHC I-binding prediction algorithms. Notably, despite the use of only MHC I-binding prediction algorithms, numerous neoantigen vaccine trials have found that several individuals immunizing peptides elicit both a CD8+ and CD4+ T-cell response;^1–3,6^ however, the significance of this remains unclear.

In this study, we found that several therapeutic neoantigen vaccines targeting an MHC I-restricted neoepitope require a conjoined T-cell helper epitope in order to engender a therapeutic CD8+ T-cell response. The reliable identification of effective helper epitopes within neoantigens remains a challenge in the clinic; therefore, we asked whether the universal helper epitope from tetanus toxin, P30, could effectively fulfill this role. Our results show that conjoining P30 to immunodominant MHC I-restricted neoepitopes was able to promote therapeutic, neoantigen-specific CD8+ T-cell responses. Remarkably, we found instances where conjoining P30 to MHC I-restricted neoepitopes that were, otherwise, poorly immunogenic (i.e., subdominant) was able to enhance immune and antitumor responses compared with neoantigen vaccines spanning the native neoantigen sequence. Together, these data suggest that the rational design of neoantigen vaccines using a universal helper epitope can improve responses to therapeutically relevant MHC I-restricted neoepitopes and provide encouraging insights for clinical neoantigen vaccine design, especially for tumors exhibiting a low mutation load.

## Results

### An effective neoantigen vaccine targeting mutant Odc1 requires a cytotoxic CD8+ and helper CD4+ T-cell response

**Fig. 1:**
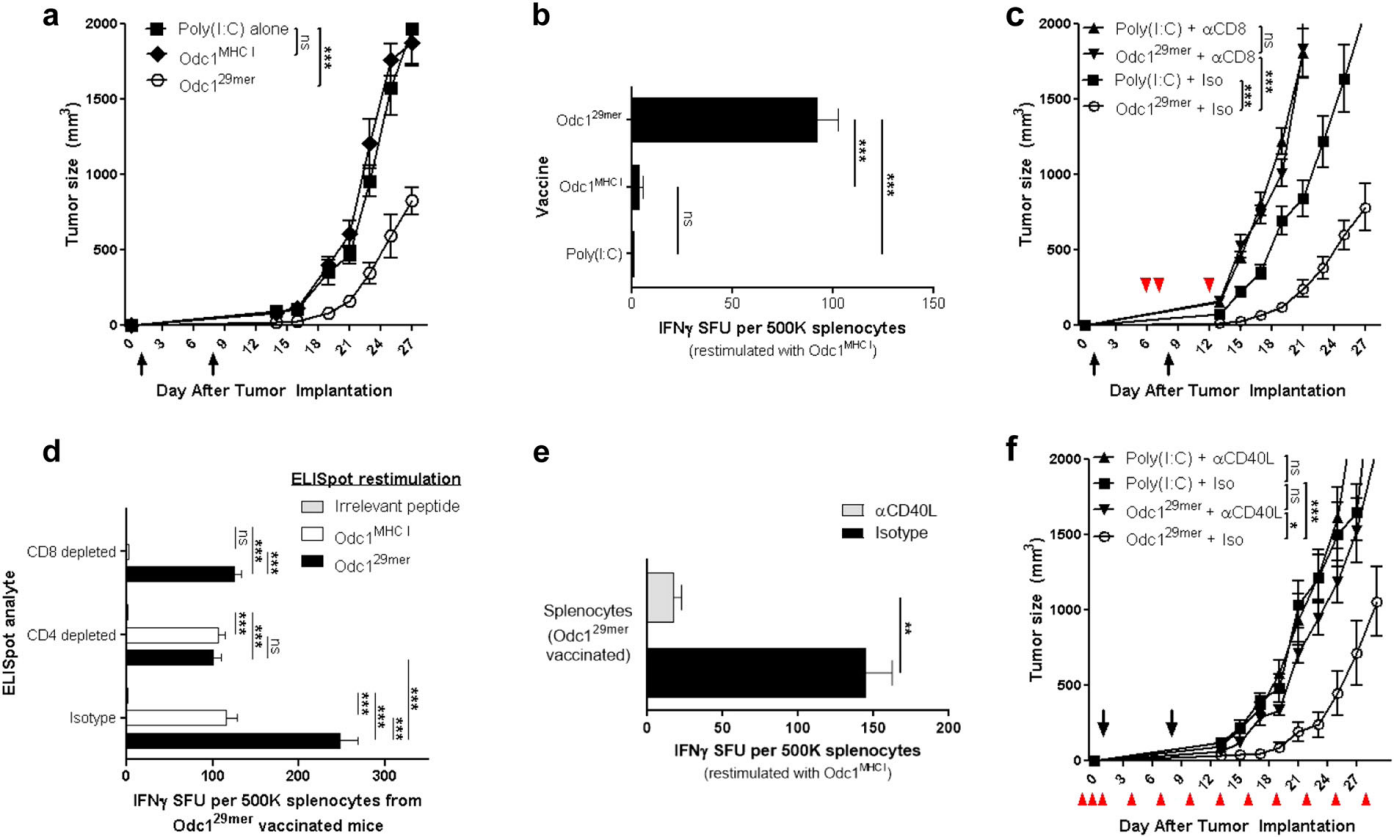
An endogenous helper epitope facilitates the MHC I neoepitope-mediated therapeutic effects of the Odc1 SLP vaccine. **a** Subcutaneous SMA560 tumor growth in mice (*n* = 7) following therapeutic immunization on days 1 and 8 with poly(I:C) alone, Odc1^MHC I^, or Odc1^29*mer*^ SLP. **b** IFNγ ELISpot: splenocyte response to Odc1^MHC I^ 7 days following immunization with poly(I:C) alone, Odc1^MHC I^, or Odc1^29*mer*^ SLP (*n* = 3). One-way ANOVA with post hoc Tukey’s test. **c** Subcutaneous SMA560 tumor growth in mice (*n* = 7) following therapeutic immunization on days 1 and 8 with poly(I:C) alone or Odc1^29*mer*^ SLP in the context of CD8+ depleting antibody or isotype control (antibody administration denoted by arrowheads). **d** IFNγ ELISpot: Odc1^29*mer*^-mediated immune response to the Odc1^MHC I^-restricted neoepitope or 29mer immunizing peptide in the context of CD8-depletion, CD4-depletion, or isotype-treated control (*n* = 3). Two-way ANOVA with Bonferroni post hoc test. **e** IFNγ ELISpot: splenocyte response to Odc1^MHC I^ 7 days following immunization with Odc1^29*mer*^ SLP in the context of CD40L-blocking antibody or isotype control (*n* = 3). Isotype vs. αCD40L, two-sample *t* test. **f** Subcutaneous SMA560 tumor growth in mice (*n* = 7) following therapeutic immunization on days 1 and 8 with poly(I:C) alone or Odc1^29*mer*^ in the context of CD40L-blocking antibody or isotype control (antibody administration denoted by arrowheads). For tumor growth data, error bars = mean ± s.e.m.; for ELISpot data, errors bars = mean ± s.d.

Upon subcutaneous or intracerebral implantation of the malignant mouse astrocytoma, SMA560 into syngeneic VMDk mice, IFNγ+CD8+ T cells recognizing an MHC I-restricted neoepitope within the neoantigen Odc1 (Odc1^MHC I^) spontaneously infiltrate the tumor^8^, suggesting that this neoepitope would serve as a model vaccine target. We, therefore, evaluated the therapeutic effects of the Odc1^MHC I^ peptide vaccine, as well as a 29mer synthetic long peptide (SLP) vaccine spanning the native Odc1 neoantigen sequence (Odc1^29*mer*^) that includes Odc1^MHC I^ (Supplementary Table 1). The latter was tested due to the preferential processing and presentation of longer peptides by professional APCs^9^. Immunization with the Odc1^29*mer*^ SLP vaccine, but not the Odc1^MHC I^ peptide vaccine, mediated significant antitumor effects against SMA560 tumors in a therapeutic setting (Fig. 1a). In addition, ELISpot analysis revealed that the Odc1^29*mer*^ SLP vaccine, but not the Odc1^MHC I^ peptide vaccine, elicited a robust IFNγ+ response towards the minimal Odc1 MHC I-restricted neoepitope, indicative of an Odc1^MHC I^-specific CD8+ T-cell response^8^, and elimination of these cells through CD8+ depletions at the effector phase abrogated the antitumor effect (Fig. 1b, c), suggesting that the antitumor effects of the Odc1^29*mer*^ SLP vaccine are mediated by CD8+ Odc1^MHC I^-specific cytotoxic lymphocytes (CTLs).

Although peptide length alone can facilitate enhanced CTL responses^9^, some SLP-mediated CTL responses are enhanced by an endogenous MHC II-restricted epitope^10^. To characterize the T-cell response generated by the Odc1^29*mer*^ vaccine, CD8+ and CD4+ splenocytes from mice immunized 7 days prior were depleted immediately before performing ELISpot analysis. CD8+ depletion confirmed that the Odc1^29*mer*^ vaccine induced a robust IFNy+CD8+ Odc1^MHC I^-specific T-cell response; however, CD8+ depletion did not eliminate the entirety of the IFNy+ response following Odc1^29*mer*^ peptide restimulation. This remaining IFNy+Odc1^29*mer*^-specific response was abolished upon depletion of CD4+ splenocytes (Fig. 1d), indicating the presence of an immunogenic MHC II-restricted epitope within the Odc1^29*mer*^ SLP.

CD4+ T cells can augment CD8+ T-cell responses by licensing dendritic cells (DCs) to become potent activators of CTLs. This mechanism, known as CD4+ T-cell help, is mediated by the engagement of CD40L on activated CD4+ T cells and CD40 on DCs^11^. Disruption of this interaction using in vivo antibody-mediated CD40L blockade dramatically suppressed the ability of Odc1^29*mer*^ SLP vaccine to induce a robust IFNγ+Odc1^MHC I^ CTL response and prevented antitumor efficacy (Fig. 1e, f). Furthermore, in vivo blockade of MHC II similarly diminished the IFNγ+Odc1^MHC I^ CTL response by the Odc1^29*mer*^ SLP vaccine (Supplementary Fig. 1), suggesting that CD40L was bestowed by the CD4+ T-cell compartment. Collectively, these data demonstrate that the Odc1^29*mer*^ SLP vaccine contains an immunogenic MHC I- and MHC II-restricted neoepitopes that induce an Odc1^MHC I^ CTL response and helper CD4+ T-cell response, respectively, and blockade of any of these responses abrogates the antitumor effects of this vaccine.

### A conjoined universal helper epitope provides the necessary T-cell help to neoepitope-specific CTL responses

**Fig. 2:**
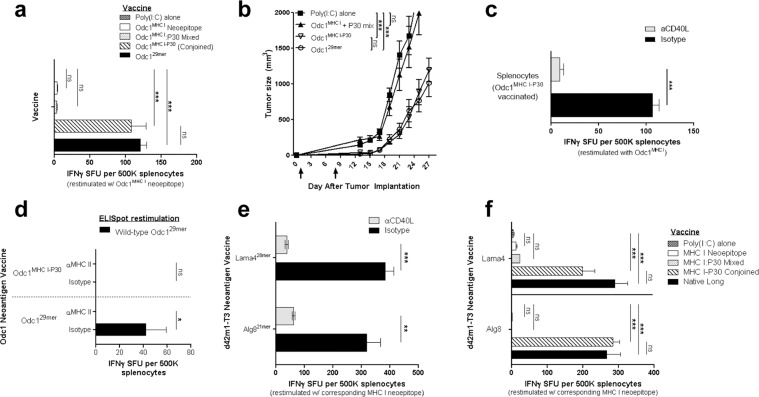
Conjoined P30 can effectively supplant the endogenous helper epitope within highly immunogenic neoantigen vaccines. **a** IFNγ ELISpot: mice (*n* = 3) were immunized with poly(I:C) alone, Odc1^MHC I^, Odc1^MHC I^ mixed with the P30 helper epitope, Odc1^MHC I^ conjoined to the P30 helper epitope, or the Odc1^29*mer*^ SLP. Seven days later, the splenocyte response to Odc1^MHC I^ was evaluated. One-way ANOVA with post hoc Tukey’s test. **b** Subcutaneous SMA560 tumor growth in mice (*n* = 7) following therapeutic immunization on days 1 and 8 with poly(I:C) alone, Odc1^MHC I^ mixed with P30, Odc1^MHC I^ conjoined to P30 (Odc1^MHC I-P30^), or the Odc1^29*mer*^ SLP. **c** IFNγ ELISpot: splenocyte response to Odc1^MHC I^ 7 days following immunization with the Odc1^MHC I-P30^ SLP in the context of CD40L-blocking antibody or isotype control (*n* = 3). Isotype vs. αCD40L, two-sample *t* test. **d** IFNγ ELISpot: splenocyte response to the wild-type 29mer peptide assessed 7 days following immunization with the Odc1^29*mer*^ or Odc1^MHC I-P30^ SLP. The contribution of MHC II to the Odc1 wild-type response determined by immunologically blocking MHC II prior to peptide restimulation (*n* = 3). Isotype vs. αMHC II, two-sample *t* test. **e** IFNγ ELISpot: splenocyte response to the Lama4 or Alg8 MHC I-restricted neoepitope 7 days following immunization with the corresponding SLP in the context of CD40L-blocking antibody or isotype control (*n* = 3). Isotype vs. αCD40L, two-sample *t* test. **f** IFNγ ELISpot: for each neoantigen, mice (*n* = 3) were immunized with poly(I:C) alone, the MHC I-restricted neoepitope alone, the MHC I-restricted neoepitope mixed with the P30 helper epitope, the MHC I-restricted neoepitope conjoined to the P30 helper epitope, or the efficacious SLP spanning the endogenous neoantigen sequence (native long). Seven days later, the splenocyte response to the corresponding MHC I-restricted neoepitope was evaluated. One-way ANOVA with post hoc Tukey’s test. For tumor growth data, error bars = mean ± s.e.m.; for ELISpot data, errors bars = mean ± s.d.

Similar to the Odc1^29*mer*^ SLP vaccine, in two recent phase I trials evaluating neoantigen vaccines in GBM patients, si of the seven (~86%) detectable neoepitope-specific IFNγ+CD8+ T-cell responses were accompanied by an IFNγ+CD4+ T-cell response to the same immunizing SLP^1,2^, implicating the clinical significance of CD4+ T-cell help in the immunogenicity of MHC I-restricted neoepitopes. The deliberate linkage of an immunogenic MHC II-restricted helper epitope to an MHC I neoepitope is an often-overlooked aspect of clinical neoantigen vaccine design. Due to the promiscuous binding of peptides to MHC II molecules, in silico MHC II-binding-prediction algorithms are currently less-accurate than prediction algorithms for MHC I^12^. These tools are also limited by their inability to gauge a host’s CD4+ T-cell precursor frequency reactive to a putative MHC II epitope, which can jeopardize an adequate helper response. Furthermore, not every immunogenic MHC I-restricted neoepitope may be in close proximity to an immunogenic MHC II-restricted epitope. Therefore, we asked whether T-cell helper function could be effectively provided by using a universally immunogenic helper epitope. For this question, we utilized the highly immunogenic supertope P30 from tetanus toxin, which (1) contains MHC II-restricted epitopes that bind to the majority of MHC II haplotypes, (2) leverages population-wide T-cell memory to tetanus toxin, providing a sufficient pool of CD4+ T-cell precursors, and (3) has been shown to be safe in humans^13–15^. When P30 and Odc1^MHC I^ were administered into mice as separate peptides, no substantial IFNγ Odc1^MHC I^ CTL response or antitumor effects were observed (Fig. 2a, b). However, a vaccine comprised of P30 conjoined to Odc1^MHC I^ as a single peptide (Odc1^MHC I-P30^) generated an IFNγ+Odc1^MHC I^ CTL response and an antitumor effect comparable to that of the Odc1^29*mer*^ SLP vaccine (Fig. 2a, b), indicating that these two elements must by physically connected. Congruent with the role of P30 as a helper epitope, CD40L blockade suppressed the ability of Odc1^MHC I-P30^ to induce a substantial IFNγ+Odc1^MHC I^ CTL response (Fig. 2c). Moreover, the immune-stimulating effects of conjoined P30 were not simply a function of increased peptide length, evidenced by the fact that conjoining Odc1^MHC I^ to a non-immunogenic peptide equivalent in length as P30 elicited no detectable IFNγ+Odc1^MHC I^ CTL response. We also found that Odc1^MHC I-P30^ prevented the induction of CD4+ T cells that cross-react with the wild-type Odc1^29*mer*^ peptide, which we have observed with the Odc1^29*mer*^ SLP (Fig. 2d).

Given the generalizable nature of neoantigen vaccine design using a universal helper epitope, we explored these principles with immunodominant MHC I neoepitopes from a non-glioma mouse tumor model. An earlier independent study identified two MHC I-restricted neoepitopes within the neoantigens Lama4 and Alg8 that were spontaneously immunogenic upon implantation of the d42m1-T3 sarcoma, and SLP vaccines spanning these MHC I-restricted neoepitopes generated a pronounced neoepitope-specific IFNγ+CD8+ CTL response that effectively treated tumors^16^. As with the Odc1^29*mer*^ SLP vaccine, we found that in vivo blockade of CD40L dramatically suppressed the ability of the Lama4 and Alg8 SLP vaccines to engender robust neoepitope-specific IFNγ+ CTL responses (Fig. 2e), further implicating the requirement for CD40L-mediated T-cell help in the therapeutic effects of efficacious neoantigen vaccines. Moreover, P30 was able to supply the requisite helper function to both Lama4 and Alg8 CTL responses but only when conjoined to the corresponding MHC I-restricted neoepitope (Fig. 2f). Together, these results demonstrate that conjoining P30 to an immunogenic MHC I-restricted epitope represents a broadly applicable and effective means for ensuring the provision of CD40L-mediated T-cell help to a neoepitope-specific CD8+ CTL response.

### A universal helper epitope can augment the therapeutic effects of neoantigen vaccines

**Fig. 3:**
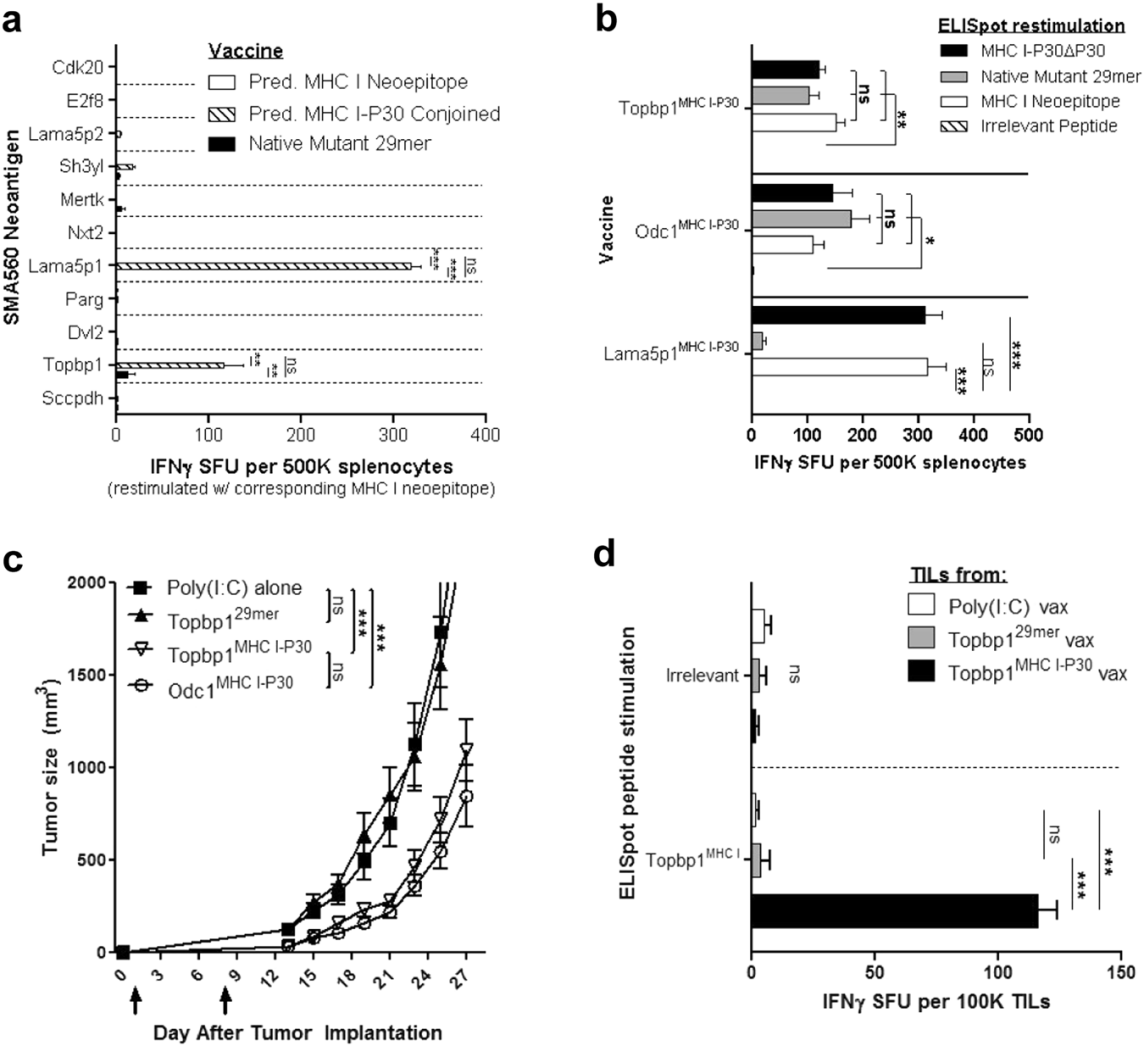
Conjoined P30 can improve the therapeutic effects of neoantigen vaccines. **a** IFNγ ELISpot: splenocyte response to the corresponding putative MHC I-restricted neoepitope 7 days following immunization with the predicted MHC I-restricted neoepitope alone, the predicted MHC I-restricted neoepitope conjoined to P30, or the 29mer SLP spanning the endogenous neoantigen sequence (*n* = 3). One-way ANOVA with post hoc Tukey’s test. **b** IFNγ ELISpot: mice (*n* = 3) were immunized with Topbp1^MHC I-P30^, Odc1^MHC I-P30^, or Lama5^MHC I-P30^ in order to induce a neoepitope-specific CD8+ T-cell response. The ability of the MHC I neoepitope, MHC I-P30, or the native 29mer peptide to restimulate neoepitope-specific CD8+ T cells was assessed 7 days following immunization. The response to P30 alone was subtracted from the MHC I-P30 response (MHC I-P30ΔP30). One-way ANOVA with post hoc Tukey’s test. **c** Subcutaneous SMA560 tumor growth in mice (*n* = 7) following therapeutic immunization on days 1 and 8 with poly(I:C) alone, Topbp1^29*mer*^, Topbp1^MHC I-P30^, or Odc1^MHC I-P30^ SLP. **d** IFNγ ELISpot: evaluation of Topbp1^MHC I^-reactive TILs within day 27 subcutaneous SMA560 tumors (*n* = 5-6) from poly(I:C), Topbp1^29*mer*^, or Topbp1^MHC I-P30^ vaccinated mice (cells alone-background subtracted). One-way ANOVA with post hoc Tukey’s test. For tumor growth data, error bars = mean ± s.e.m.; for ELISpot data, errors bars = mean ± s.d.

Thus far, we have evaluated the effects of conjoined P30 on MHC I-restricted neoepitopes that are highly immunogenic, reflected by the spontaneous recruitment of neoepitope-cognate IFNγ+CD8+ T cells into untreated tumors^8,16^. We next wished to determine the effects of conjoined P30 on MHC I-restricted neoepitopes that are intrinsically poorly immunogenic, that is those that are not naturally immunogenic upon tumor implantation. To this end, we first assessed the reactivity of tumor-infiltrating lymphocytes (TILs) isolated from untreated SMA560 tumors to 12 top-predicted MHC I-restricted neoepitopes (IEDB MHC I percentile rank score ≤0.4; Supplementary Table 1), which included Odc1^MHC I^, using the IFNγ ELISpot assay. Only IFNγ+ TILs reactive to Odc1^MHC I^ were identified (Supplementary Fig. 2). P30-conjoined vaccines were, therefore, generated for the remaining 11 putative MHC I-restricted neoepitopes that were not naturally immunogenic and evaluated for their immunogenicity. Nine of the 11 (~82%) P30-conjoined vaccines did not elicit a pronounced (i.e., IFNγ+ SFU > 20) immune response to their respective putative MHC I-restricted neoepitope. Remarkably, however, vaccines composed of P30 conjoined to the H-2K^b^-binding neoepitopes Lama5p1 (Lama5p1^MHC I-P30^) or Topbp1(Topbp1^MHC I-P30^) induced a robust neoepitope-specific CD8+ T-cell response that far exceeded the corresponding response engendered by the native 29mer peptide vaccine possessing the MHC I-restricted neoepitope (Fig. 3a, Supplementary Fig. 3).

Despite the ability of the Lama5p1^MHC I-P30^ vaccine to generate a strong Lama5p1^MHC I^-specific CD8+ T-cell response, these cells could not be restimulated with the native Lama5p1^29*mer*^ peptide (Fig. 3b), suggesting that Lama5p1^MHC I^ may not be sufficiently processed from the endogenous neoantigen and subsequently presented (i.e., not antigenic). Consistent with this hypothesis, antitumor effects were not observed upon therapeutic immunization with the Lama5p1^MHC I-P30^ vaccine (Supplementary Fig. 4a), and IFNγ+ Lama5p1^MHC I^-specific TILs were not detected in SMA560 tumors harvested from Lama5p1^MHC I-P30^-vaccinated mice (Supplementary Fig. 4b). Alternatively, Topbp1^MHC I^-specific CD8+ T cells could be restimulated with the native Topbp1^29*mer*^ peptide (Fig. 3b), suggesting antigenicity. Correspondingly, the Topbp1^MHC I-P30^ vaccine elicited a significant antitumor benefit that was associated with the infiltration of IFNγ+ Topbp1^MHC I^-specific TILs into the tumor. Strikingly, neither antitumor efficacy nor infiltration of IFNγ+ Topbp1^MHC I^-specific TILs into tumors was observed upon immunization with the native Topbp1^29*mer*^ SLP (Fig. 3c, d). Taken together, these results demonstrate that the rational design of SLPs in which a predicted MHC I-restricted neoepitope is conjoined to the universal helper epitope P30 can render neoantigen vaccines more immunogenic and efficacious compared to SLPs that span the native neoantigen sequence.

## Discussion

Neoantigen-specific T cells play a critical role in driving antitumor immunity. Their appeal for immunotherapy stems from their ability to exclusively target tumor cells, which grants a mitigated risk of off-target effects. This feature is especially important when dealing with tumors arising in vital organs, such as the brain and central nervous system tissues^17^. Nevertheless, conventional neoantigen vaccine strategies have struggled to induce robust CD8+ T-cell responses directed towards a patient’s repertoire of putative MHC I-restricted neoepitopes.

Our work highlights the importance of designing a neoantigen vaccine to contain both an MHC I-restricted neoepitope and a conjoined MHC II-restricted helper epitope in order to render a productive CD4+ T-cell helper response, which is required for the priming, memory formation, and maintenance of highly cytolytic CD8+ T cells in chronic pathologies, such as cancer^18^. These findings are consistent with a recent study showing that a vaccine comprised of irradiated tumor cells expressing two neoantigens, one containing an immunogenic MHC I-restricted neoepitope and the other containing an immunogenic MHC II-restricted helper neoepitope, engendered superior antitumor effects compared with a vaccine consisting of a mixture of irradiated tumor cells expressing each neoantigen alone^19^. However, our data suggest that T-cell helper function can be effectively provided to a neoantigen-specific CD8+ T-cell response by using a conjoined heterologous helper epitope.

Neoantigen vaccine design using a universally immunogenic heterologous helper epitope is a simple and clinically tractable strategy that circumvents several challenges associated with predetermining MHC class II helper epitopes from neoantigens, including the moderate fidelity of MHC II-binding prediction algorithms^12^ and defining a host’s neoepitope-specific CD4+ T-cell precursors. Moreover, conventional long neoantigen vaccines spanning the native neoantigen sequence possess significant homology with the wild-type antigen, and as a result, they often induce a wild-type reactive CD4+ T-cell response^1–3,20,21^. Given the well-described role of autoreactive CD4+ T cells in central nervous system autoimmune pathologies^17^, these effects should be minimized, if possible. Vaccines composed of a minimal MHC I-restricted neoepitope conjoined to a foreign helper epitope naturally limit the region that is homologous with the wild-type antigen, thereby mitigating the activation of autoreactive CD4+ T cells, as we have observed with neoantigen vaccines targeting Odc1. In this study, we utilized the P30 helper epitope from tetanus toxin in light of its widespread immunogenicity in humans^15^. A serological survey showed that ~70% or more Americans have immunity to tetanus^22^, making an assessment of P30-specific CD4+ T-cell memory feasible in a clinical setting. Alternatively, it is likely that other foreign helper epitopes with widespread immunogenicity, such as PADRE, could also be safely used for this purpose^23^.

A salient finding from this study is that a conjoined universal helper epitope can unveil immune and antitumor responses to poorly immunogenic, subdominant MHC I-restricted neoepitopes, evidenced by our findings with Topbp1-targeting neoantigen vaccines. Several tumor types, such as GBM^24^, exhibit a high degree of intratumoral heterogeneity, resulting in differential antigen expression from cell to cell. In these instances, while a given tumor may possess highly immunogenic MHC I-restricted neoepitopes, it is unlikely that these mutations would be ubiquitously expressed throughout the tumor burden. Thus, immunotherapeutic strategies that can unmask immune responses to subdominant neoepitopes, thereby expanding the repertoire of therapeutically relevant MHC I-restricted neoepitopes that are expressed throughout the tumor burden, would have clear advantages for antigenically heterogeneous tumors.

A major challenge of vaccine design, in general, is the predetermination of epitopes that will be recognized by the host’s adaptive immune system. The most basic requirements of an immunotherapeutically relevant MHC I-restricted neoepitope is one for which the host possesses an adequate pool of cognate CD8+ T-cell precursors and one that is sufficiently processed from the underlying antigen and presented to the immune system on MHC I molecules. As a result of the spontaneous mechanisms of cancer immunoediting, immunodominant MHC class I neoepitopes can sometimes be identified by analyzing a patient’s peripheral blood or TIL compartment^25^; however, subdominant neoepitopes may not be detectable in this manner, as we have observed with Topbp1. This may be less of an issue, with respect to vaccine design, for tumors with a low mutational load (e.g., primary GBM) since it may be practical to immunize against all top-predicted MHC I neoepitopes, but prioritizing potential subdominant MHC I neoepitopes would require more rigorous consideration for tumors with a high mutational burden. For this purpose, analytical approaches that empirically assess neoepitope binding to MHC I molecules may prove useful. Examples of this include mass spectrometric analysis of neopeptides bound to tumor-derived MHC I molecules or the use of MHC I-matched transporter associated with antigen processing-deficient cells (e.g., T2) to identify neoepitopes with a high peptide:MHC I-binding affinity.

Although we observed modest antitumor benefits against subcutaneous SMA560 tumors with Odc1^MHC I-P30^ or Topbp1^MHC I-P30^ vaccines using a simple prime-boost vaccine regimen, antitumor effects were not achieved against intracerebrally implanted SMA560 tumors. Central nervous system (CNS)-resident tumors are protected from the immune system, to some extent, by semi-restrictive blood-CNS barriers and the lack of a pronounced lymphatic network. Nevertheless, we and others have detected Odc1-specific CD8+ T cells within untreated intracerebral SMA560 tumors in similar amounts to that of subcutaneous SMA560 tumors^8^, signifying a functional immunological network between the peripheral immune system and brain tumors. Of note, the recruitment of neoantigen-specific T cells into brain tumors is not idiosyncratic to mouse models, as clinical studies have demonstrated an influx of these cells into GBM tumors upon immunization with neoantigen vaccines^1,2^. These results suggest that approaches that enhance the functionality of neoepitope-specific T cells may augment the therapeutic effects of neoantigen vaccines against brain tumors. Consistent with this hypothesis, we have previously found that SIINFEKL-specific CD8+ T cells are present in untreated, intracerebrally implanted B16 melanoma tumors expressing ovalbumin (B16OVA)^26^, but a vaccine composed of SIINFEKL conjoined to P30 (SIINFEKL-P30) is ineffective, on its own, against intracerebral B16OVA tumors—analogous to our findings with the Odc1 neoantigen in SMA560. However, when SIINFEKL-P30 is administered in conjunction with an agonist antibody targeting the T-cell costimulatory molecule CD27, a significant survival benefit against intracerebral B16OVA tumors is observed^13^. Additional supporting evidence has come from another study showing that coadministration of anti-PDL1 antibody is capable of unleashing the antitumor effects of a polyvalent neoantigen vaccine against intracerebral CT2A^27^, an aggressive mouse brain tumor. Collectively, these data suggest that neoantigen vaccines alone will likely be insufficient to treat orthotopic human gliomas, but the effects of these vaccines against intracerebral tumors may be improved by using combination therapies that modulate immune checkpoints.

## Methods

### Cell lines, mice, and media

All animal experiments were performed in accordance with Duke University’s Institutional Animal Care and Use Committee-approved protocol. Wild-type VMDk mice were bred in-house under pathogen-free conditions at Duke University Medical Center. Male 129S6 mice were acquired from Taconic Farms. For immunogenicity and tumor studies, 6–12-week-old mice were used. SMA560 tumor cells were cultured in IMEM-Zinc Option medium (Gibco) containing 10% fetal bovine serum (FBS) (Gemini Bio-Products) and 1× penicillin–streptomycin (Gibco). R10 medium consisted of RPMI (Gibco), 10% FBS, 1× MEM-NEAA (Gibco), and 1× penicillin–streptomycin. T-cell medium consisted of R10 medium with 1 mM glutamine (Gibco), 1 mM sodium pyruvate (Gibco), 50 μM beta-mercaptoethanol (Gibco), and 100 U/mL IL-2. Fluorescence-activated cell sorting (FACS) buffer consisted of 1× PBS (Gibco) plus 2% FBS (Gemini Bio-Products).

### Next-generation exome sequencing and ribosome profiling

Exome libraries were captured from SMA560 tumor cells and syngeneic healthy VMDk brain tissue using the SureSelect Mouse All Exon kit (Agilent). Sequencing was performed on an Illumina HiSeq2000 as 100 bp paired-end reads, and the resulting reads were aligned to the mm9 mouse reference genome. Variants within the protein-coding sequence were called using the Illumina CASAVA pipeline. Neoantigen expression was determined by ribosome profiling^28^. To this end, 10^7^ SMA560 cells were washed thrice with ice-cold PBS and then lysed on ice for 5 min in 600 μL lysis buffer containing 20 mM Tris-HCl, 150 mM KCl, 10 mM MgCl_2_, and 1% NP-40. Clarified supernatant containing polysomes was treated with micrococcal nuclease (New England BioLabs) and mRNA fragments purified using TRIzol^TM^ reagent (Invitrogen) and isopropanol precipitation. RNA pellets were treated with T4 polynucleotide kinase (New England BioLabs) followed by electrophoresis through a 15% TBE-urea polyacrylamide gel, using a 35 nt oligo (IDT) as a reference. The ~35 nt mRNA footprint was carefully excised from the gel, crushed, and precipitated with 400 mM sodium acetate. Polyacrylamide was removed by centrifuging samples through a 0.45 μM SPIN-X column (Corning). Following ethanol precipitation, the mRNA fragments were ligated into the NEBNext® Small RNA Library (New England BioLabs). Sequencing was performed using Illumina MiSeq as 50 bp single end reads. Reads were mapped to the GRCm38 reference assembly using HISAT2 and transcript abundance was determined using Cufflinks^29^. Cross-referencing the ribosome profiling results with the exome sequencing data served as an indicator of neoantigen expression within SMA560. Genes with an FPKM > 0.25 were regarded as “expressed” and included in downstream studies. The expression of mutated Odc1, Lama5, and Topbp1 within SMA560 cells was confirmed using RT-PCR and Sanger sequencing.

### Bioinformatic prioritization of putative neoepitopes

Putative MHC I-restricted neoepitopes flanking the 225 expressed missense mutations were assessed with Immune Epitope Database (IEDB) MHC I prediction algorithms^30^ using a 29mer peptide sequence spanning the neoantigen sequence with the mutation at residue 15. Predicted MHC I-restricted neoepitopes were ranked by their IEDB percentile rank score.

### SLP vaccines

All peptides were synthesized by Genscript. The sequences of all tested SMA560 29mer SLPs, with the mutation at residue 15, are listed in Supplementary Table 1. The peptide sequences used for Lama4 and Alg8 vaccines were QKISFFDGFE**VGFNFRTL**QPNGLLFYYT and AVG**ITYTWTRL**YASVLTGSLV (MHC I-restricted neoepitope underlined), respectively^16^. For P30-conjugated vaccines, furin-P30 (RVKRFNNFTVSFWLRVPKVSASHLE) was positioned after the carboxyl terminus of the MHC I-restricted neoepitope.

### RMA-S MHC class I stabilization assay

RMA-S cells were rested at 26 °C/5% CO_2_ in R10 medium for 24 h and then wash once with 1× PBS (Gibco). RMA-S cells (10^6^) were resuspended in R10 medium containing 10 μM peptide, and incubated at 26 °C for 3 h and then at 37 °C for 1 h. SIINFEKL served as H-2K^b^ positive control, hgp100 as H-2D^b^-positive control, and DMSO was a negative control. Cells were then stained in FACS buffer containing APC-anti-H-2Kb (clone AF6-88.5.5.3, eBioscience) and PE-anti-H-2Db (clone 28-14-8, eBioscience) at 4 °C for 30 min, washed twice, and then analyzed by flow cytometry.

### In vivo immune and tumor studies

For tumor studies, three days prior to tumor implantation, the right rear flank of VMDk mice was shaved. Mice were inoculated with 7.5 × 10^5^ SMA560 tumor cells suspended in 1× PBS (Gibco). Tumor growth was determined by taking orthogonal measurements using a digital caliper. Tumor volume was calculated using the formula “length × width^2^ × 0.52”. Mice were killed if a tumor measurement surpassed 2 cm or reached a total volume of 2000 mm^3^. To determine the contribution of CD8+ cells in the Odc1^29*mer*^-mediated antitumor effect, 200 μg αCD8a (clone 2.43, BioXCell) or the corresponding isotype (BioXCell) antibody was administered intraperitoneally on days 6, 7, and 12 following tumor implantation. In other immunogenicity studies, VMDk mice were given 300 μg αMHC Class II (clone M5/114, BioXCell), or 200 μg αCD40L (clone MR-1, BioXCell), or the corresponding isotype (BioXCell) antibody intraperitoneally on days—1, 0, and 2 relative to a vaccine. 129S6 mice received 400 μg αCD40L or isotype antibody, owing to greater spleen cellularity. All experiments were repeated at least three times.

### TIL processing

To isolate TILs for ELISpot analysis, ~100 mg excised tumor was minced into small pieces in 15 mL of T-cell medium and transferred into a paddle blender bag (standard size, 105 ×155 mm). Tumor was dissociated in a paddle blender (Stomacher® 80 Biomaster, Seward) for 45 min at 37 °C. Dissociated tissue was then passed through a 70 μm cell strainer into 50 mL conical tube. Following centrifugation at 350 g for 10 min, cells were resuspended in 40 mL T-cell medium and 20 mL seeded into two T150 flasks. Flasks were incubated for 12 h at 37 °C/ 5% CO_2_ in order to remove adherent cells. Nonadherent cells were harvested by pouring off, resuspended in 15 mL fresh T-cell medium, and replated into a T75 flask. Cells were incubated at 37 °C/ 5% CO_2_ for an additional 2.5 days before harvesting for ELISpot analysis^26^.

### IFNγ ELISpot assay

Splenocytes or TILs were filtered using a 70 μM strainer. Cells were resuspended in 8 mL R10 medium, layered onto Lympholyte-M (Cedarlane), and isopycnic centrifugation performed at 1300 × *g* for 25 min with no brake. The buffy coat layer was removed and cells washed twice with R10 medium before counting using a flow cytometer (Guava easyCyte, Millipore Sigma). In all, 5 × 10^5^ splenocytes or 1 × 10^5^ TILs were plated per well of a 96-well polyvylidene difluoride (plates (Multiscreen HTS, Millipore Sigma) that had previously been coated with 10 μg mL^−1^ anti-IFNγ antibody (clone AN18, Mabtech). Cells were stimulated with 1 μM peptide or 4 μg mL^−1^ ConA. After an 18–22 h incubation period at 37 °C/5% CO_2_, IFNγ secretion was detected using 1 μg mL^−1^ biotinylated anti-IFNγ (clone R4-6A2 antibody, Mabtech) followed by development with avidin-conjugated peroxidase (Vectastain ABC, Vector Laboratories) and AEC (Sigma). For MHC II-blocking ELISpots, 20 μg mL^−1^ anti-MHC II antibody (M5/114, BioXcell) was incubated with cells for 2 h prior to peptide stimulation. For bead-based depletion ELISpots, CD4+ or CD8+ cells were removed by adding 6 × 10^6^ antibodies (clone 53-6.7, CD8; clone GK1.5, CD4; Rat IgG2 κ, isotype, BioLegend)-conjugated beads (M280 Dynabeads, Invitrogen) in 10 μLs to each sample, gently rotated for ~8 min, and beads magnetically removed. For TIL and cell-depleted ELISpots, 2.5 × 10^4^ naive splenocytes were added to each well to provide a source of APCs, as a precaution. All samples were evaluated in duplicate.

### Statistical analysis

ELISpot data were collected from distinct mice and evaluated using an unpaired two-sample *t* test or one-way analysis of variance with post hoc Tukey’s test^31^. For tumor growth curves, repeated measures were taken from the same mice. A mixed-effects linear model was employed utilizing log-transformed tumor measurements; *F* tests were conducted to compare the rate of tumor growth over time (i.e., regression line slopes). An autoregressive correlation structure was assumed among the repeated measures within an animal. Asterisks indicate degree of significance (*P < 0.05, **P ≤ 0.01, ***P < 0.001, *P* > 0.05 not significant (ns)).

### Reporting summary

Further information on research design is available in the Nature Research Reporting Summary linked to this article.

## Supplementary information

Supplementary Information

Reporting Summary

## Data Availability

The data that support the findings of this study are available from the corresponding author upon reasonable request. This study did not generate any unique code.
