# Supplementary material for: A conjoined universal helper epitope can unveil antitumor effects of a neoantigen vaccine targeting an MHC class I-restricted neoepitope
Source: NPJ Vaccines. 2021 Jan 18;6:12. doi: 10.1038/s41541-020-00273-5 (PMC7814002; doi:10.1038/s41541-020-00273-5)
Supplement: Supplementary file 1 — Supplementary Information [file 41541_2020_273_MOESM1_ESM.pdf]

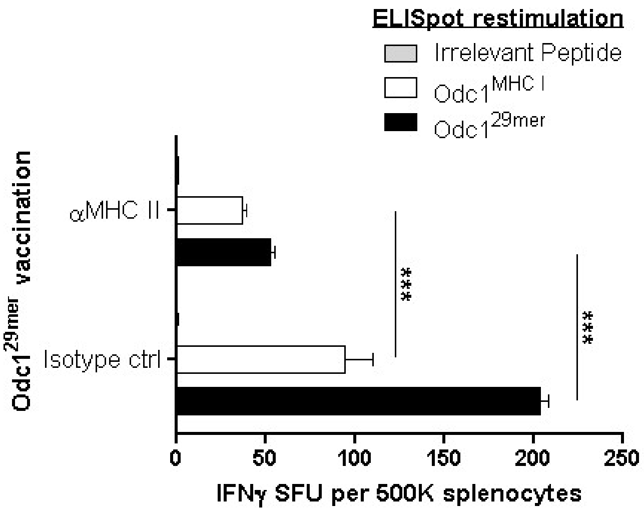

Supplementary Figure 1:
In vivo blockade of MHC II suppresses the Odc1^{29mer} SLP vaccine-mediated CTL response. IFNγ ELISpot: splenocyte response to Odc1$^{MHC\ I}$ or Odc1^{29mer} 7 days following immunization with Odc1^{29mer} in the context of MHC II-blocking antibody or isotype control (n = 3). Two-way ANOVA with Bonferroni post-hoc test. Errors bars = mean ± s.d.

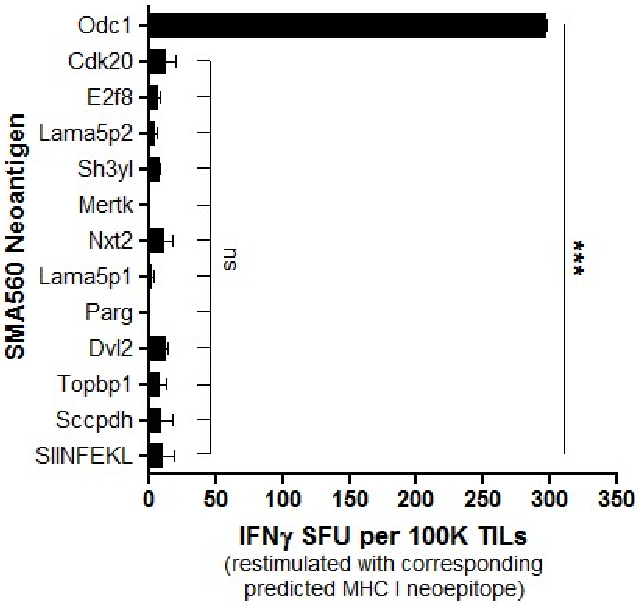

Supplementary Figure 2:

Odc1$^{MHC\ I}$-reactive TILs detected within untreated SMA560 tumors. IFNγ ELISpot: TILs from untreated day 27 subcutaneous (left, n =2) SMA560 tumors were evaluated for their reactivity to predicted MHC I-restricted neoepitopes (IEDB MHC I percentile rank score ≤ 0.4). Responses were compared to stimulation with the negative-control peptide SIINFKEL. One-way ANOVA with post-hoc Tukey's test. Representative data of two independent experiments. Errors bars = mean ± s.d.

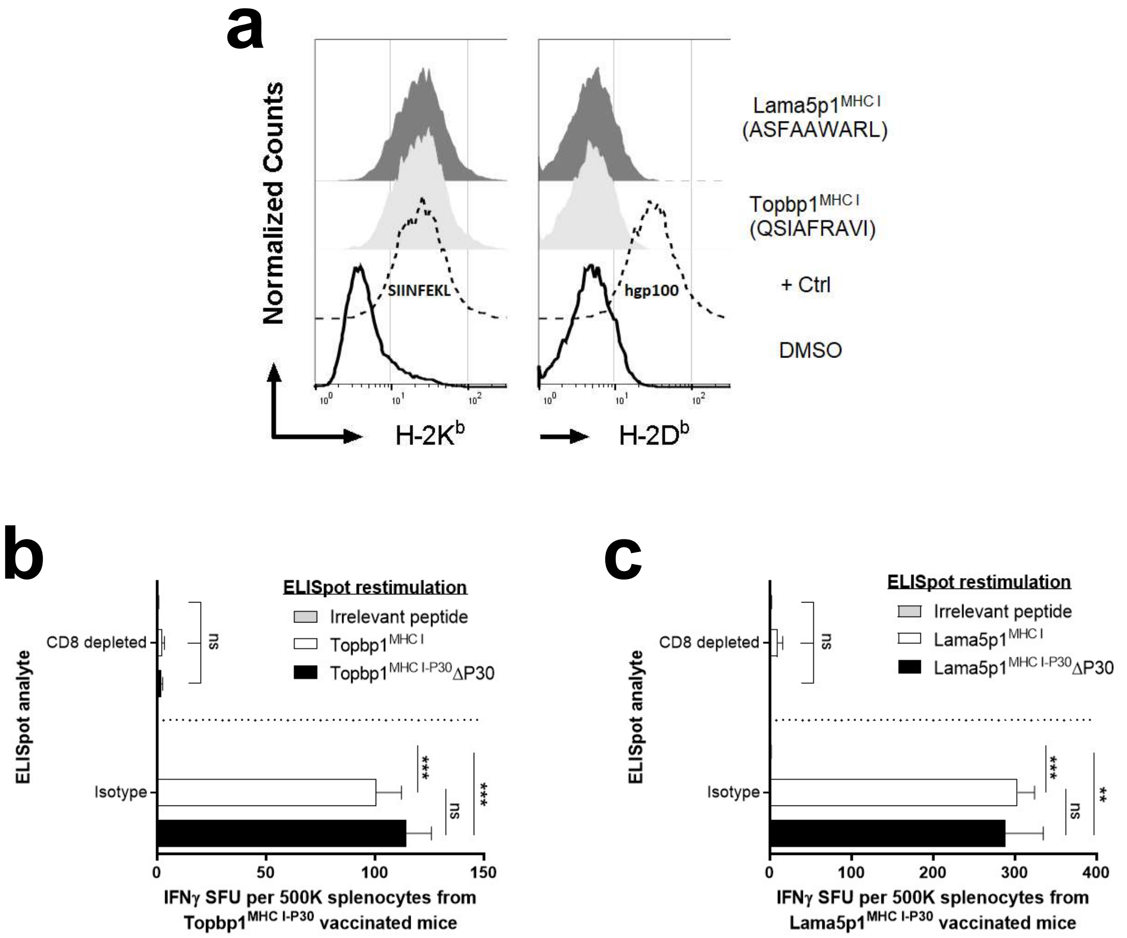

Supplementary Figure 3:
Lama5p1$^{\text{MHC I}}$ and Topbp1$^{\text{MHC I}}$ bind H-2K^{b} and induce a cognate CD8+ T-cell response when conjoined to P30. (**a**) MHC I stabilization of Lama5p1$^{\text{MHC I}}$ and Topbp1$^{\text{MHC I}}$ on TAP-deficient RMA-S cells. (**b**) IFNγ ELISpot: Topbp1$^{\text{MHC I-P30}}$-mediated and (**c**) Lama5p1$^{\text{MHC I-P30}}$-mediated immune response to their respective MHC I-restricted neoepitope and MHC I-P30 peptide evaluated 7 days after immunization (n = 3). Splenocytes were treated with isotype or CD8-depleting antibody-coated beads prior to loading ELISpot. The response to P30 alone was subtracted from the MHC I-P30 response (MHC I-P30ΔP30). Two-way ANOVA with Bonferroni post-hoc test. Errors bars = mean ± s.d.

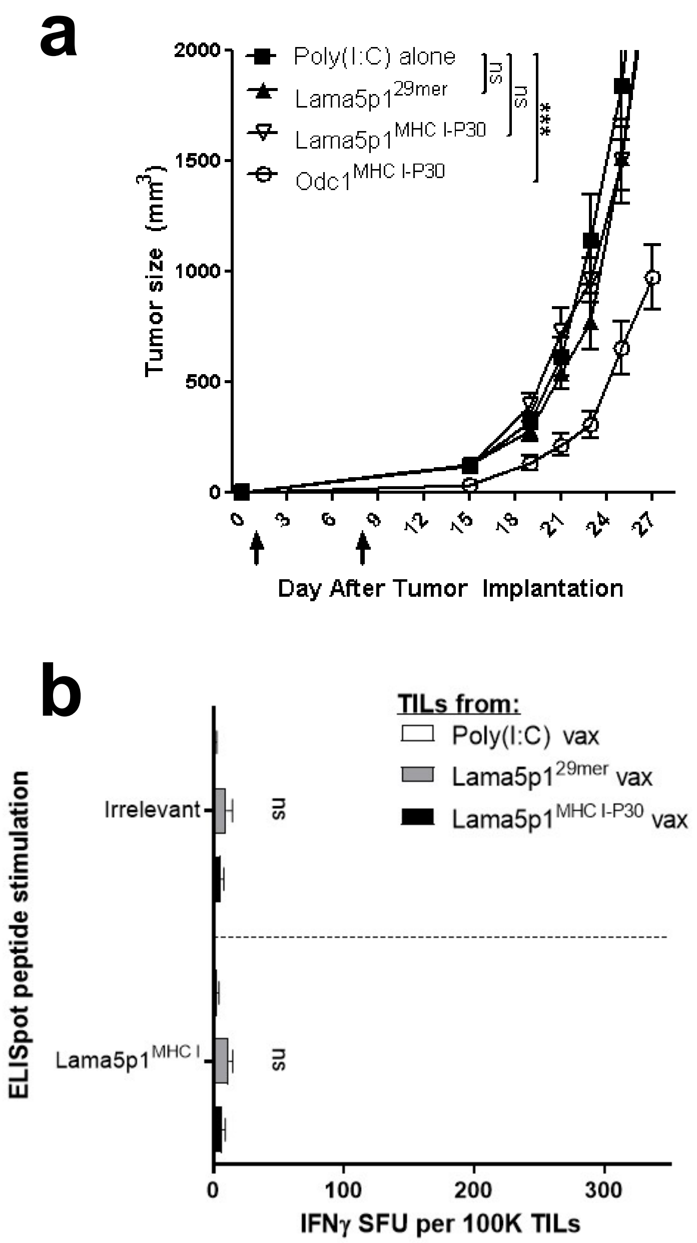

Supplementary Figure 4:
Lama5p1$^{MHC\ I\text{-}P30}$ does not elicit antitumor effects or promote infiltration of Lama5p1$^{MHC\ I}$-specific T cells into SMA560 tumors. (**a**) Subcutaneous SMA560 tumor growth in mice (n = 7) following therapeutic immunization on days 1 and 8 with poly(I:C) alone, Lama5p1^{29mer}, Lama5p1$^{MHC\ I\text{-}P30}$, or Odc1$^{MHC\ I\text{-}P30}$ SLP. (**b**) IFNγ ELISpot: evaluation of Lama5p1$^{MHC\ I}$-reactive TILs within day 27 subcutaneous SMA560 tumors (n = 6) from poly(I:C), Lama5p1^{29mer}, or Lama5p1$^{MHC\ I\text{-}P30}$ vaccinated mice (cells alone-background subtracted). One-way ANOVA with post-hoc Tukey's test. For tumor growth data, error bars = mean ± s.e.m.; for ELISpot data, errors bars = mean ± s.d.

Supplementary Table 1. Top 12 predicted MHC I-restricted neoepitopes from neoantigens expressed in SMA560

GeneID	FPKM[1]	MutResidue	AA From	AA to	IEDB[2] MHC I PR[3]	Predicted MHC I Neoepitope	Pred IC50 (ANN)	Pred IC50 (SMM)	Native 29mer SLP Vaccine[4]
Cdk20	0.806392	87	D	Y	0.15	LAFEFMLS**Y**L	66.33	1205.95	HGAGFVLAFEFMLS**Y**LAEVVRHAQRPLAP
E2f8	0.866666	272	K	R	0.15	MSQ**R**FVML	10.7	18.52	VNSRKDKSLRVMSQ**R**FVMLFLVSTPQIVS
Lama5p2	1.13509	897	G	V	0.15	VRF**V**FNPL	6.42	9.59	LEEAATPEGHAVRF**V**FNPLEFENFSWRGY
Odc1	2.50028	129	Q	L	0.2	YAASNGV**L**M	9.09	58.29	KQVSQIKYAASNGV**L**MMTFDSEIELMKVA
Sh3yl1	0.340289	100	D	Y	0.2	VS**Y**LVIIL	21.36	30.67	AGLGGGFEIGIEVS**Y**LVIILNYDRAVEAF
Mertk	1.51034	682	R	P	0.2	HTFLLYS**P**L	13.08	13.42	FMKYGDLHTFLLYS**P**LNTGPKYIHLQTLL
Nxt2	2.90821	109	F	V	0.2	H**V**FNQNFL	144.33	4029.49	VTSGVVKFDGNKQH**V**FNQNFLLTAQSTPN
Lama5p1	1.13509	2201	S	F	0.3	AS**F**AAWARL	12.17	112.49	LPAIREQLQGINAS**F**AAWARLHRLNASIA
Parg	2.97412	516	V	L	0.3	CSEQNLYP**L**	89.19	125.49	KHVKMPCSEQNLYP**L**EDENGERTAGSRWE
Dvl2	1.26082	385	A	T	0.4	AWVSHSA**T**L	186.01	147.27	IQPIDPAAWVSHSA**T**LTGAFPAYPGSSSM
Topbp1	1.84552	792	R	I	0.4	QS**I**AFRAVI	30.29	95	KKAVTPLDMNRFQS**I**AFRAVISQQRGQDP
Sccpdh	6.39732	380	Q	H	0.4	IAMV**H**AAM	17.78	103.93	GPEAGYVATPIAMV**H**AAMTFLSDASDLPK

[1]Fragments per kilobase of transcript per million mapped reads

[2]Immune Epitope Database

[3]Percentile rank

[4]Missense mutation bolded. Predicted MHC I-restricted neoepitope underlined.
