# Supplementary material for: A conjoined universal helper epitope can unveil antitumor effects of a neoantigen vaccine targeting an MHC class I-restricted neoepitope
Source: NPJ Vaccines. 2021 Jan 18;6:12. doi: 10.1038/s41541-020-00273-5 (PMC7814002; doi:10.1038/s41541-020-00273-5)
Supplement: Supplementary file 2 — Reporting Summary [file 41541_2020_273_MOESM2_ESM.pdf]

nature research

Corresponding author(s): John H. Sampson

Last updated by author(s): Nov 27, 2020

Reporting Summary

Nature Research wishes to improve the reproducibility of the work that we publish. This form provides structure for consistency and transparency in reporting. For further information on Nature Research policies, see our Editorial Policies and the Editorial Policy Checklist.

Statistics

For all statistical analyses, confirm that the following items are present in the figure legend, table legend, main text, or Methods section.

n/a	Confirmed	
☐	☒	The exact sample size (n) for each experimental group/condition, given as a discrete number and unit of measurement
☐	☒	A statement on whether measurements were taken from distinct samples or whether the same sample was measured repeatedly
☐	☒	The statistical test(s) used AND whether they are one- or two-sided<br>*Only common tests should be described solely by name; describe more complex techniques in the Methods section.*
☐	☒	A description of all covariates tested
☐	☒	A description of any assumptions or corrections, such as tests of normality and adjustment for multiple comparisons
☐	☒	A full description of the statistical parameters including central tendency (e.g. means) or other basic estimates (e.g. regression coefficient) AND variation (e.g. standard deviation) or associated estimates of uncertainty (e.g. confidence intervals)
☐	☒	For null hypothesis testing, the test statistic (e.g. F, t, r) with confidence intervals, effect sizes, degrees of freedom and P value noted<br>*Give P values as exact values whenever suitable.*
☒	☐	For Bayesian analysis, information on the choice of priors and Markov chain Monte Carlo settings
☒	☐	For hierarchical and complex designs, identification of the appropriate level for tests and full reporting of outcomes
☒	☐	Estimates of effect sizes (e.g. Cohen's d, Pearson's r), indicating how they were calculated

Our web collection on statistics for biologists contains articles on many of the points above.

Software and code

Policy information about availability of computer code

Data collection	No software was used for data collection
Data analysis	MHC I binding predictions were assessed using the browser-based Immune Epitope DataBase MHC-I Binding Prediction tool version 2.22

For manuscripts utilizing custom algorithms or software that are central to the research but not yet described in published literature, software must be made available to editors and reviewers. We strongly encourage code deposition in a community repository (e.g. GitHub). See the Nature Research guidelines for submitting code & software for further information.

Data

Policy information about availability of data

All manuscripts must include a data availability statement. This statement should provide the following information, where applicable:

- Accession codes, unique identifiers, or web links for publicly available datasets
- A list of figures that have associated raw data
- A description of any restrictions on data availability

The data that support the findings of this study are available from the corresponding author upon reasonable request.

Field-specific reporting

Please select the one below that is the best fit for your research. If you are not sure, read the appropriate sections before making your selection.

☒ Life sciences ☐ Behavioural & social sciences ☐ Ecological, evolutionary & environmental sciences

For a reference copy of the document with all sections, see nature.com/documents/nr-reporting-summary-flat.pdf

Life sciences study design

All studies must disclose on these points even when the disclosure is negative.

Sample size	For tumor efficacy experiments, sample sizes were chosen based on subcutaneous SMA560 growth patterns we have repeatedly observed using this model. For immunogenicity experiments, sample sizes were chosen based on our extensive experience using peptide vaccines and the ELISpot assay to determine these outcomes in mice.
Data exclusions	No data was excluded.
Replication	All immunogenicity and antitumor studies were repeated at least 3 times to ensure reproducibility.
Randomization	Six to 12 week old mice were used for all immunogenicity and tumor efficacy experiments. For each experiment, mice were randomized by placing all experimental mice in a single cage and randomly selecting mice for each group.
Blinding	Blinding was not relevant to this study

Reporting for specific materials, systems and methods

We require information from authors about some types of materials, experimental systems and methods used in many studies. Here, indicate whether each material, system or method listed is relevant to your study. If you are not sure if a list item applies to your research, read the appropriate section before selecting a response.

Materials & experimental systems

n/a	Involved in the study	
☐	☒	Antibodies
☐	☒	Eukaryotic cell lines
☒	☐	Palaeontology and archaeology
☐	☒	Animals and other organisms
☒	☐	Human research participants
☒	☐	Clinical data
☒	☐	Dual use research of concern

Methods

n/a	Involved in the study	
☒	☐	ChIP-seq
☒	☐	Flow cytometry
☒	☐	MRI-based neuroimaging

Antibodies

Antibodies used	For ELISpot analysis: anti- mouse IFNγ antibody (clone AN18, Mabtech), biotinylated anti-mouse IFNγ (clone R4-6A2 antibody, Mabtech), anti-mouse MHC II antibody (M5/114, BioXcell), anti-mouse CD8 (clone 53-6.7, Biolegend), anti-mouse CD4 (clone GK1.5)<br><br>For in vivo depletion/blocking experiments: anti-mouse CD8a (clone 2.43, BioXCell), αnti-mouse MHC Class II (clone M5/114, BioXCell), anti-mouse CD40L (clone MR-1, BioXCell)
Validation	All primary antibodies were shown to bind to primary antigenic target by the vendor.

Eukaryotic cell lines

Policy information about cell lines

Cell line source(s)	SMA560 cells were derived from VMDK mice at Duke University by Dr. Darell Bigner
Authentication	We had our stock of SMA560 cells authenticated by IDEXX
Mycoplasma contamination	We had IDEXX confirm that our SMA560 stock was free of mycoplasma contamination
Commonly misidentified lines (See ICLAC register)	n/a

Animals and other organisms

Policy information about studies involving animals; ARRIVE guidelines recommended for reporting animal research

Laboratory animals	Six to 12 week old male VMDK and 129S6 mice were used for these studies
Wild animals	Wild animals were not involved in these studies.
Field-collected samples	This study did not involve samples collected from the field.
Ethics oversight	Duke IACUC approved all animal protocols for this study

Note that full information on the approval of the study protocol must also be provided in the manuscript.
